# Validity of an Artificial Intelligence-Based Application to Identify Foods and Estimate Energy Intake Among Adults: A Pilot Study

**DOI:** 10.1016/j.cdnut.2023.102009

**Published:** 2023-09-29

**Authors:** Chloe P. Lozano, Emma N. Canty, Sanjoy Saha, Stephanie T. Broyles, Robbie A. Beyl, John W. Apolzan, Corby K. Martin

**Affiliations:** 1 Pennington Biomedical Research Center, Baton Rouge, LA, United States; 2 University of Hawaii at Manoa, Honolulu, HI, United States

**Keywords:** energy intake, artificial intelligence, identification, dietary assessment, pilot study, validity, mobile application

## Abstract

**Background:**

The commercial application Openfit allows for automatic identification and quantification of food intake through short video capture without a physical reference marker. There are no known peer-reviewed publications on the validity of this Nutrition Artificial Intelligence (AI).

**Objectives:**

To test the validity of Openfit to identify food automatically and semiautomatically (with user correction), test the validity of Openfit at quantifying energy intake (kcal) automatically and semiautomatically, and assess satisfaction and usability of Openfit.

**Methods:**

During a laboratory-based visit, adults (7 male and 17 female), used Openfit to automatically and semiautomatically record provided meals, which were covertly weighed. Foods logged were identified as an “exact match,” “far match,” or an “intrusion” using Food and Nutrient Database for Dietary Studies (FNDDS) codes. Descriptive data were stratified by meal, food item, and FNDDS group, and presented with or without beverages. Bland–Altman analyses assessed errors over levels of energy intake. Participants completed a User Satisfaction Survey (USS) and the Computer Systems Usability Questionnaire (CSUQ). Open-ended questions were assessed with qualitative methods.

**Results:**

Exact matches, far matches, and intrusions were 46%, 41%, and 13% for automated identification, and 87%, 23%, and 0% for semiautomated identification, respectively. Error for automated and semiautomated energy estimates were 43% and 33% with beverages, and 16% and 42% without beverages. Bland–Altman analyses indicated larger error for higher energy meals. Overall mean scores were 2.4 for the CSUQ and subscale means scores ranged from 4.1 to 5.5. for the USS. Participants recommended improvements to Openfit’s Nutrition AI, manual estimation, and overall app.

**Conclusion:**

Openfit worked relatively well for automatically and semiautomatically identifying foods. Error in automated energy estimates was relatively high; however, after excluding beverages, error was relatively low (16%). For semiautomated energy estimates, error was comparable to previous studies. Improvements to the Nutrition AI, manual estimation and overall application may increase Openfit’s usability and validity.

This trial was registered at clinicaltrials.gov as NCT05343585.

## Introduction

Technology-based tools reduce the burden of completing a dietary record and are often preferred over traditional assessment methods, e.g., paper-based food records [[1], [2], [3]]. Dietary assessment applications can also provide real-time feedback on nutrient intake, which may assist with behavior and dietary change [1,4,5]. Mobile applications are popular for tracking food intake. Based on a national United States survey conducted in 2017, findings revealed that 14% of adults in the United States reported consistent usage of a mobile application for diet and nutrition tracking. Additionally, 17% of them used such applications occasionally, 11% had employed an application at least once, 39% expressed their openness to using an application for diet and nutrition tracking, whereas only 19% indicated that they would not opt for an app-based dietary assessment method [6]. Approximately 91% of dietary assessment applications created for research purposes and only 30% of applications designed for commercial use have been evaluated through published peer-reviewed validity studies [1]. This highlights the need for further validation of commercially available dietary tracking applications to ensure accurate reporting of dietary intake.

With advancements in technology, dietary assessment mobile applications have added functions for automated food identification and quantification. These methods and technologies are relatively new, and, to our knowledge, there are no peer-reviewed publications on automated identification and estimation of energy intake through short video capture without the use of a reference marker (e.g., a fiducial marker). Commercial applications, including Openfit, Simple app, the premium version of MyFitnessPal, and Naiya, use short video capture without a physical reference marker for automated dietary tracking; therefore, the accuracy and reliability of this technology must be evaluated. These commercial applications also allow users to manually change the automatically identified and quantified food items, but no known peer-reviewed publication has reported on the validity of these “semiautomatic” methods. To address these research gaps, the primary aims of this pilot study were as follows: *1)* to assess the validity of Nutrition AI in Openfit for automatic and semiautomatic (with user correction) food identification; *1)* to evaluate the accuracy of Nutrition AI in Openfit for automated and semiautomated quantification of energy intake; and *1)* to gauge user satisfaction and usability of Nutrition AI in Openfit among adult participants.

## Methods

### Recruitment and study design

For this pilot study, data were collected from a sample of adults (*n* = 24) residing in Louisiana. This sample size surpasses the guidelines by Hertzog [7], which recommend that 10–15 participants are typically sufficient for a pilot study. Participants were recruited by the Pennington Biomedical Research Center (PBRC) Recruitment Core through the Current Research Trials webpage and the internal PBRC listserv. Inclusion criteria were adults aged 18–62 years with a BMI between 18.5 and 50 kg/m^2^. Exclusion criteria were any condition or circumstance that could impede study completion, women who were pregnant or breastfeeding, and individuals who were unfamiliar with or unable to use a mobile application. Participants underwent an initial eligibility screening via the PBRC website, followed by a subsequent confirmation of eligibility through a telephone verification process. If eligible, participants completed one study visit at PBRC lasting ≤2 hours. During the visit, participants provided informed consent, and study staff trained participants on how to use the Openfit application to record dietary intake. Participants then recorded 2 simulated meals using the Openfit application on a study iPhone provided. Each study visit was recorded on the study iPhone using the screen recording function to capture all automatic and semiautomatic energy estimates of food logged into Openfit. While using the app, participants were encouraged to ask questions only if the application was malfunctioning (e.g., if the application screen froze) to simulate a more realistic user experience. After logging the 2 meals into Openfit, participants completed the Computer Systems Usability Questionnaire (CSUQ) [[8], [9], [10]] and a User Satisfaction Survey (USS) [11]. Participants received $50 upon completion of the study. This study was conducted in accordance with the Declaration of Helsinki, and all procedures involving human subjects were approved by the PBRC institutional review board (IRB Federal Wide Assurance: 00006218). The trial was registered on ClinicalTrials.gov (NCT05343585) before study recruitment.

### Study menus

Study menu items and kilocalorie levels were determined by the research team and included foods commonly consumed by adults in Louisiana [12]. The final menus, with energy information, were created by the PBRC Metabolic Kitchen Core using the Moore's Extended Nutrient database [13,14]. Each food item served was covertly weighed to one tenth of a gram by the PBRC Metabolic Kitchen and all other study staff were blinded to the energy content of the provided meals.

A selection of 32 distinct food items was employed to formulate 5 distinct meals: Chicken meal, Burger meal, pizza meal, Pork chop meal, and a salad meal. Subsequently, 2 variants of each meal were crafted to represent distinct energy levels spanning from 400 to 800 kcal (Supplementary Figures 1–5). For instance, the pizza meal was available in both 500 (Supplementary Figure 2) and 800 kcal (Supplementary Figure 3) options. This approach encompassed higher and lower energy alternatives for each meal, ensuring that energy content did not disproportionately influence error rates across meal types. In total, there were 10 distinct meals. Participants were assigned randomly to 2 of these 10 meals (Supplementary Figures 1–5) and were tasked with employing the application to estimate the energy content (in kilocalories) of each of the 2 meals provided.

Beverages were presented in transparent cups, whereas condiments were placed in transparent containers alongside the meals. Notably, no food items were presented in packaging to participants. A place card denoting the name of each food item was supplied to facilitate the identification process. This step was necessary as participants neither selected, prepared, nor consumed the study-provided food.

### Food identification and energy estimation

Non–peer-reviewed information about the Openfit application is available online [15,16]. Briefly, the Nutrition AI for automated identification in Openfit uses edge inference with hierarchical networking [17,18]. The food image, from the short video capture, is passed through a single shot detector (SSD) regional proposal network and bounding boxes are added to each food item [18]. These bounding boxes are then sent to the hierarchical network for more detailed recognition. The SSD produces one prediction of the food identity, and the hierarchical network produces 2 predictions, a Softmax prediction and a Cosine prediction. The SSD, Softmax, and Cosine predictions are then passed through a voting logic and the final food identification is sent to the user. The food-recognition engine for the Nutrition AI uses neural networks to identify food by recognizing key features of raw, cooked, and prepared food, and any text, the nutrition facts label, bar codes, and logos on packaged food [16].

Nutrition AI in the Openfit application uses multiple images from a video stream for structure and pose estimation, image rectification, dense stereo reconstruction, food surface extraction, and food volume estimation [18]. This volume estimation method is based on research by Puri et al. [19]. The application then converts the food volume to grams, ounces, kilocalories, and serving sizes [16] but only information on kilocalories and nutrients consumed is available to the user once food items are logged.

During the current study, participants were instructed to initially utilize the video stream (scanning feature) in the Openfit application for the automatic identification of food items and the estimation of portion sizes, both of which corresponded with kilocalories information. In instances where a participant found the automatic identification or portion size estimation to be inaccurate, they were guided to employ a manual approach. This involved searching for the correct food name and manually determining the portion size using the Openfit app, which also aligned with kilocalories information. For instance, if the Nutrition AI identified a food item as 0.5 cup of tomato salad, participants had the option to manually modify it, such as changing tomato salad to lettuce. Subsequently, using the dropdown portion options available in Openfit, participants could select the appropriate unit (e.g., cup or gram for lettuce) and input the estimated quantity accordingly.

Trained study staff allocated a Food and Nutrient Database for Dietary Studies (FNDDS) 2019–2020 code to each study food served to participants and a FNDDS code to each food logged by a participant in Openfit. FNDDS codes are 8 digits in length, with the first digit representing the major food group. The major food codes include the following: *1)* milk and milk products, *2)* meat, poultry, fish, and mixtures, *3)* eggs, *4)* dry beans, peas, other legumes, nuts, and seeds, *5)* grain products, *6)* fruits, *7)* vegetables, *8)* fats, oils, and salad dressings, and *9)* sugars, sweets, and beverages [20]. If a mixed dish was logged into Openfit as components (e.g., hamburger logged as hamburger patty, hamburger bun, or tomato), each component was allocated an FNDDS number for the food provided and an FNDDS number for the food logged by the participant.

An “exact match” was defined as a match of all 8 digits of the FNDDS codes for food provided and foods logged into Openfit (Table 1). A “far match” was defined as a match of only the first digit of FNDDS codes between foods provided and foods logged into Openfit. An item was defined as an “intrusion” when the first digit of the FNDDS codes did not match between foods provided and foods logged into Openfit. An “omission” was defined as a food item that was provided to participants but not scanned using Openfit or if the food item logged had no nutrient information available in Openfit. Food items marked as omissions were excluded from all analyses (*n* = *5* out of 260 food items).

**TABLE 1:** Examples of an exact match, far match, intrusion, and omission for FNDDS codes between food provided by study staff and food logged into Openfit by study participants

	Study food Name (FNDDS code)	Food logged into Openfit Name (FNDDS code)
Exact match	Cheeseburger (27510170)	Cheeseburger (27510170)
Far match	Cheeseburger (27510170)	Turkey burger (27545110)
Intrusion	Cheeseburger (27510170)	Croissant sandwich (58127310)
Omission	Cheeseburger (27510170)	Food items not scanned into Openfit by participants or nutrient information not available in Openfit

Abbreviation: FNDDS, United States Department of Agriculture’s Food and Nutrient Database for Dietary Studies.

For automated and semiautomated energy estimation, the exact energy amounts logged in the Openfit application were transferred into an electronic file for comparison with weighed food estimates. Therefore, the automated and semiautomated energy estimates in the Openfit application were generated from the nutrient database used by Openfit; however, there is no published information on this nutrient database.

### Study questionnaires

An online study eligibility screening questionnaire was used to collect self-reported age, gender, height, and weight. During the study visit, using Research Electronic Data Capture (REDCap) [21,22] on a study laptop, participants completed a demographics questionnaire, the CSUQ, and a USS. REDCap is a secure, Web-based application designed to support data capture for research studies, providing: *1)* an intuitive interface for validated data entry; *2)* audit trails for tracking data manipulation and export procedures; *3)* automated export procedures for seamless data downloads to common statistical packages; and *4)* procedures for importing data from external sources [22]. The USS was adapted from a previous version used to assess the usability of a mobile application for dietary assessment [11]. The USS included 5 quantitative questions and 3 open response questions. The quantitative questions were scored using a 6-point Likert scale, with higher scores being more favorable. Quantitative questions were used to assess satisfaction, ease of use, and adequacy of training on how to use the Openfit app. The CSUQ is frequently used to assess the usability of mobile applications [23,24]. The CSUQ consists of 19 questions, each scored using a 7-point Likert scale (lower scores being more favorable), and participants rated satisfaction, usefulness, information quality, and interface quality of the Openfit app. The average of these 19 questions provides an overall usability score.

### Statistical analysis

To present findings related to automated and semiautomated identification and energy intake estimation, we report descriptive statistics, stratified by meal level, food item level, FNDDS food groups, and the inclusion or exclusion of beverages in the analyses. For the analyses without beverages, the beverages removed for these analyses were 1% milk from the milk and milk products FNDDS group, and Coca Cola, Diet Coke, and sweet tea from the sugars, sweets, and beverages FNDDS group. For food identification, descriptive data are provided for exact matches, far matches, and intrusions. Data were assessed for normality and no transformations were required. Paired test of proportions was used to test for significant difference in food items identified as an exact match, far match, and intrusion between automated and semiautomated estimates. To achieve a minimum power of 80% for detecting differences in proportions of 30% or greater, a total of 40 food items were required. To assess >40 food items across meals, 24 participants were needed to provide automated and semiautomated estimates for 2 meals each. For energy intake, mean (±SD) kcal, percent error, and estimates within ± 10% kcal and ± 25% kcal are provided to allow for comparisons between weighed food items, automated estimates, and semiautomated estimates. The ±25% kcal bounds were selected to align with the appropriate statistical power for a pilot study [11,25]. Employing bounds of ±10% kcal allowed for more stringent assessment of error estimates, although power was limited for these analyses. Percent error was calculated as [(automated or semiautomated kcal – weighed kcal)/weighed kcal] ∗100. At the meal level, mean error, Bland–Altman analyses, and general linear models were performed to determine errors in automated and semiautomated estimates of energy intake with and without beverages. Quantitative data for the CSUQ and USS were averaged across participants. All quantitative statistical analyses were performed using IBM SPSS software (version 28.0.1; IBM Corporation). Open-ended responses for the USS were evaluated using qualitative methods to identify common themes [26]. Responses were independently coded by 2 researchers (CL and EC). Each coder identified preliminary codes and themes and then met to decide on the final codes and codebook. Responses were then reanalyzed by the 2 coders using the codebook, and final themes were determined.

## Results

Of the 38 people screened for the study through the PBRC website, 33 were eligible. Of those screened as eligible, 24 were scheduled for a study visit and no participants dropped out. Therefore, 24 participants (17 female and 7 male) completed the study (Table 2). Most participants were White (70.8%), and all participants had some college education or greater. The mean (± SD) age was 35.0 (± 9.5) years and the mean (± SD) BMI was 24.6 (± 4.1) kg/m^2^.

**TABLE 2:** Participant characteristics^1^

Characteristics	*n* (%)
Gender	
Male	7 (29.2)
Female	17 (70.8)
Race	
African American	4 (16.7)
Asian	3 (12.5)
White	17 (70.8)
Education	
Some college or Bachelor’s degree	10 (41.7)
Postgraduate degree	14 (58.3)
Employment	
Full-time	22 (91.7)
Part-time	2 (8.3)

	Mean ± SD	Range
Age (y)	35.0 ± 9.5	24.0–53.0
Body mass index (kg/m^2^)	24.6 ± 4.1	18.7–35.5

1 Self-reported data from the REDCap demographics questionnaire.

### Food identification

For automated identification, across all meals, 46% (118/255) of food items logged into Openfit were an exact match to food provided, 41% (105/255) were a far match, and 13% (13/255) were intrusions. The pizza meal had the lowest percentage of food items that were an exact match, which was 27% (13/49) (Table 3). The hamburger meal had the highest percentage of food items that were an exact match, which was 67% (32/48). For the salad meal, Pork chop meal, and Chicken meal, the percentages of food items that were an exact match were 40% (23/57), 45% (22/49), and 54% (28/52), respectively. Far matches ranged from 23% (11/48) for the hamburger meal to 59% (29/49) for the pizza meal. Intrusions ranged from 9% (5/57) for the salad meal to 16% (8/49) for the Pork chop meal.

**TABLE 3:** Frequency of food items correctly identified through automated (Nutrition AI) and semiautomated (Nutrition AI with user adjustment) methods, by meal type

	*n* Food Items	Exact^1^			Far^2^			Intrusion^3^		*P*^4^
		Automated	Semiautomated	*P*^4^	Automated	Semiautomated	*P*^4^	Automated	Semiautomated	
Chicken meal	52	28 (54)	47 (90)	<0.001	17 (33)	5 (10)	<0.001	7 (13)	0 (0)	0.008
Hamburger meal	48	32 (67)	47 (98)	<0.001	11 (23)	1 (2)	0.002	5 (10)	0 (0)	0.025
Pizza meal	49	13 (27)	40 (82)	<0.001	29 (59)	9 (18)	<0.001	7 (14)	0 (0)	0.008
Pork chop meal	49	22 (45)	41 (84)	<0.001	19 (39)	8 (16)	<0.001	8 (16)	0 (0)	0.005
Salad meal	57	23 (40)	46 (81)	<0.001	29 (51)	11 (19)	<0.001	5 (9)	0 (0)	0.025

Data presented as *n* (%).

1 Exact match (all 8 digits match).

2 Far match (first digit matches).

3 No match with the Food and Nutrient Database for Dietary Studies (FNDDS) food code. Food items marked as an omission were excluded, *n* = 5 items.

4 Difference between automated and semiautomated identification using a paired test of proportions.

For semiautomated identification, across all meals, 87% (221/255) of food items logged into Openfit were an exact match to food items served, 23% (34/255) of items were a far match, and there were no intrusions. Exact matches were lowest for the salad meal, which was 81% (46/57) and highest for the hamburger meal, which was 98% (47/48) (Table 3). Far matches ranged from 1% (2/48) for the Burger meal to 11% (19/57) for the salad meal, and there were no intrusions. Across each meal, exact matches, far matches, and number of intrusions significantly improved (all *P* values < 0.025) once the user corrected (semiautomated identification) the name of food automatically identified by the Nutrition AI (Table 3).

At the item level, 2% (5/260) of food items were marked as omissions and removed from all analyses. Pizza was marked as an omission on 2 occasions because no nutrient information was available in Openfit for the pizza that the participant selected. Hard butter (*n* = 1), carrots (*n* = 1), and sweet tea (*n* = 1) were marked as omissions because the participant did not scan the food using the Nutrition AI in Openfit.

After excluding beverages, for automated identification, frequencies of exact matches, far matches, and intrusions were 57% (118/209), 31% (64/209), and 13% (27/209), respectively. At the meal level, across all food items, exact matches ranged from 33% (13/39) for the pizza meal to 82% (32/39) for the hamburger meal (Table 4). Intrusions ranged from 8% (4/43) for the salad meal to 16% (7/43) for the Chicken meal. For semiautomated identification exact matches, far matches, and intrusions were 90% (188/209), 10% (21/209), and 0% (0/209), respectively. At the meal level, across all food items, exact matches ranged from 100% (39/39) for the hamburger meal to 82% (32/39) for the pizza meal, and there were no intrusions (Table 4). Except for the hamburger meal, exact matches and intrusions significantly improved after automated estimates were changed by participants (semiautomated estimates) (all *P* values < 0.05) (Table 4).

**TABLE 4:** Frequency of food items correctly identified through automated (Nutrition AI) and semiautomated (Nutrition AI with user adjustment) methods, by meal type, excluding beverages

	*n* Food Items	Exact^1^			Far^2^			Intrusion^3^		*P*^4^
		Automated	Semiautomated	*P*^4^	Automated	Semiautomated	*P*^4^	Automated	Semiautomated	
Chicken meal	43	28 (65)	40 (93)	<0.001	8 (19)	3 (7)	0.06	7 (16)	0 (0)	0.008
Hamburger meal	39	32 (82)	39 (100)	0.10	3 (8)	0 (0)	0.08	4 (10)	0 (0)	0.41
Pizza meal	39	13 (33)	32 (82)	<0.001	20 (51)	7 (18)	0.002	6 (15)	0 (0)	0.014
Pork chop meal	40	22 (55)	34 (85)	<0.001	12 (30)	6 (15)	0.014	6 (15)	0 (0)	0.014
Salad meal	43	23 (48)	43 (90)	<0.001	21 (44)	5 (10)	<0.001	4 (8)	0 (0)	0.046

Data presented as *n* (%).

1 Exact match (all 8 digits match).

2 Far match (first digit matches).

3 No match with the Food and Nutrient Database for Dietary Studies (FNDDS) food code. Food items marked as an omission were excluded, *n* = 5 items.

4 Difference between automated and semiautomated identification using a paired test of proportions.

Stratified by FNDDS food groups, for automated identification, exact matches ranged from 0% (0/37) for sugars, sweets, and beverages, to 90% (17/19) for fruits (Table 5). Far matches ranged from 10% (2/19) for fruits to 89% (33/37) for sugars, sweets, and beverages, and intrusions ranged from 0% (0/19) for fruits to 14% (37/38) for fats, oils, and salad dressings. For semiautomated identification, exact matches ranged from 50% (6/12) for milk and milk products to 97% (38/39) for grain products, far matches ranged from 3% (1/39) for grain products to 50% (6/12) for milk and milk products, and there were no intrusions (Table 5). Across all food groups, except for fruits, exact matches from the Nutrition AI significantly improved with user correction (all *P* values < 0.05) (Table 5). The food items contributing to these exact matches, far matches, and intrusions are detailed in Supplementary Table 1.

**TABLE 5:** Frequency of food items was correctly identified through automated (Nutrition AI) and semiautomated (Nutrition AI with user adjustment) methods, by the FNDDS food group

	*n* Food Items	Exact^1^			Far^2^			Intrusion^3^		*P*^4^
		Automated	Semiautomated	*P*^4^	Automated	Semiautomated	*P*^4^	Automated	Semiautomated	
Milk and milk products	12	2 (17)	6 (50)	0.046	8 (67)	6 (50)	0.16	2 (16)	0 (0)	0.16
Meat, poultry, fish and mixtures	37	19 (51)	31 (84)	<0.001	15 (41)	6 (16)	0.007	3 (8)	0 (0)	0.08
Grain products	39	27 (69)	38 (97)	<0.001	11 (28)	1 (3)	0.002	1 (3)	0 (0)	0.32
Fruits	19	17 (90)	17 (90)	1.00	2 (10)	2 (10)	1.00	0 (0)	0 (0)	1.00
Vegetables	73	50 (69)	64 (88)	<0.001	15 (20)	9 (12)	0.058	8 (11)	0 (0)	0.005
Fats, oils, and salad dressings	38	3 (8)	35 (92)	<0.001	21 (55)	3 (8)	<0.001	14 (37)	0 (0)	<0.001
Sugars, sweets, and beverages	37	0 (0)	30 (81)	<0.001	33 (89)	7 (19)	<0.001	4 (11)	0 (0)	0.046

Data presented as *n* (%).

1 Exact match (all 8 digits match).

2 Far match (first digit matches).

3 No match with the Food and Nutrient Database for Dietary Studies (FNDDS) food code. Food items marked as an omission were excluded, *n* = 5 items.

4 Difference between automated and semiautomated identification using a paired test of proportions.

### Estimation of energy intake

**FIGURE 1:**
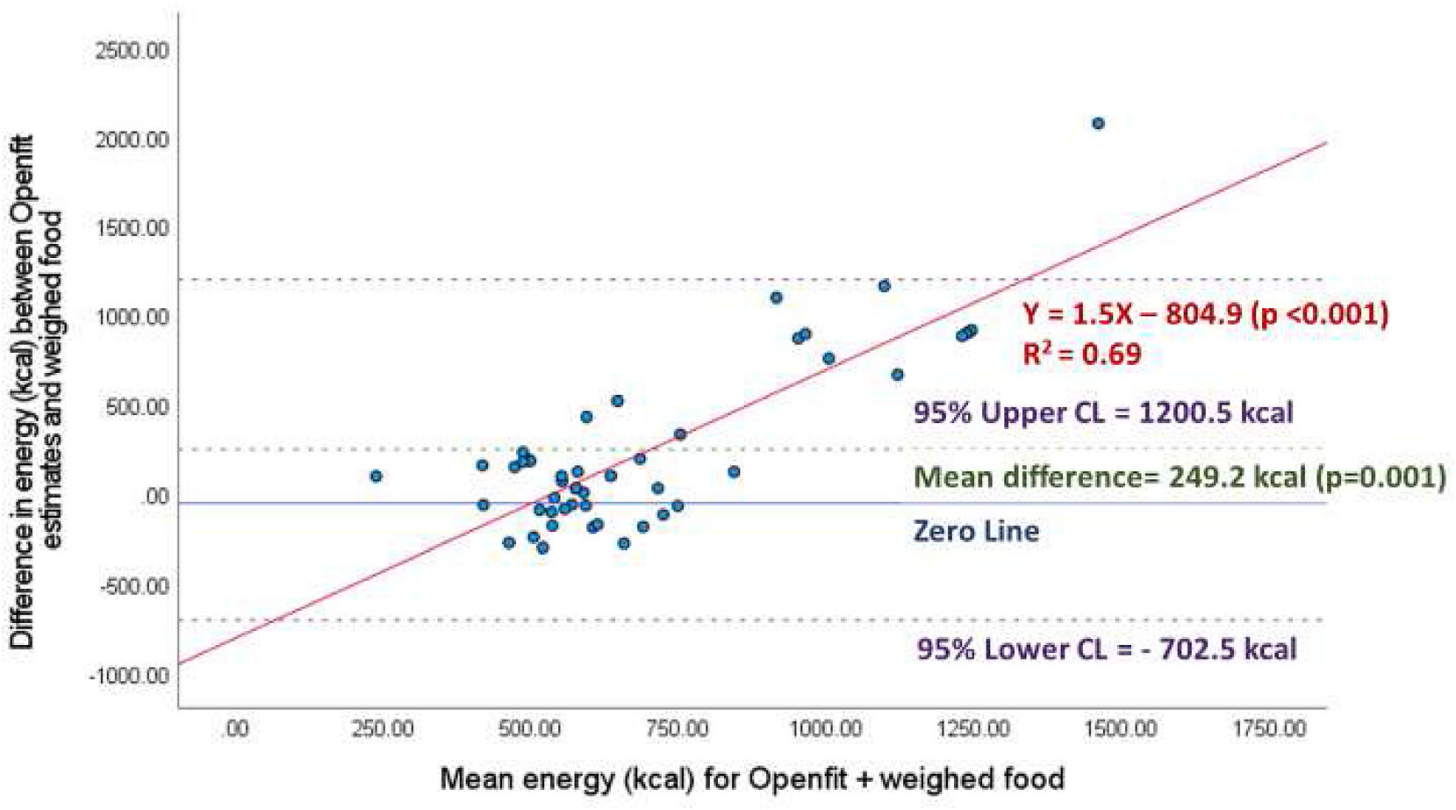
Automated estimation of energy (kcal) per meal (*n* = 48), by Nutrition AI in the Openfit app.

**FIGURE 2:**
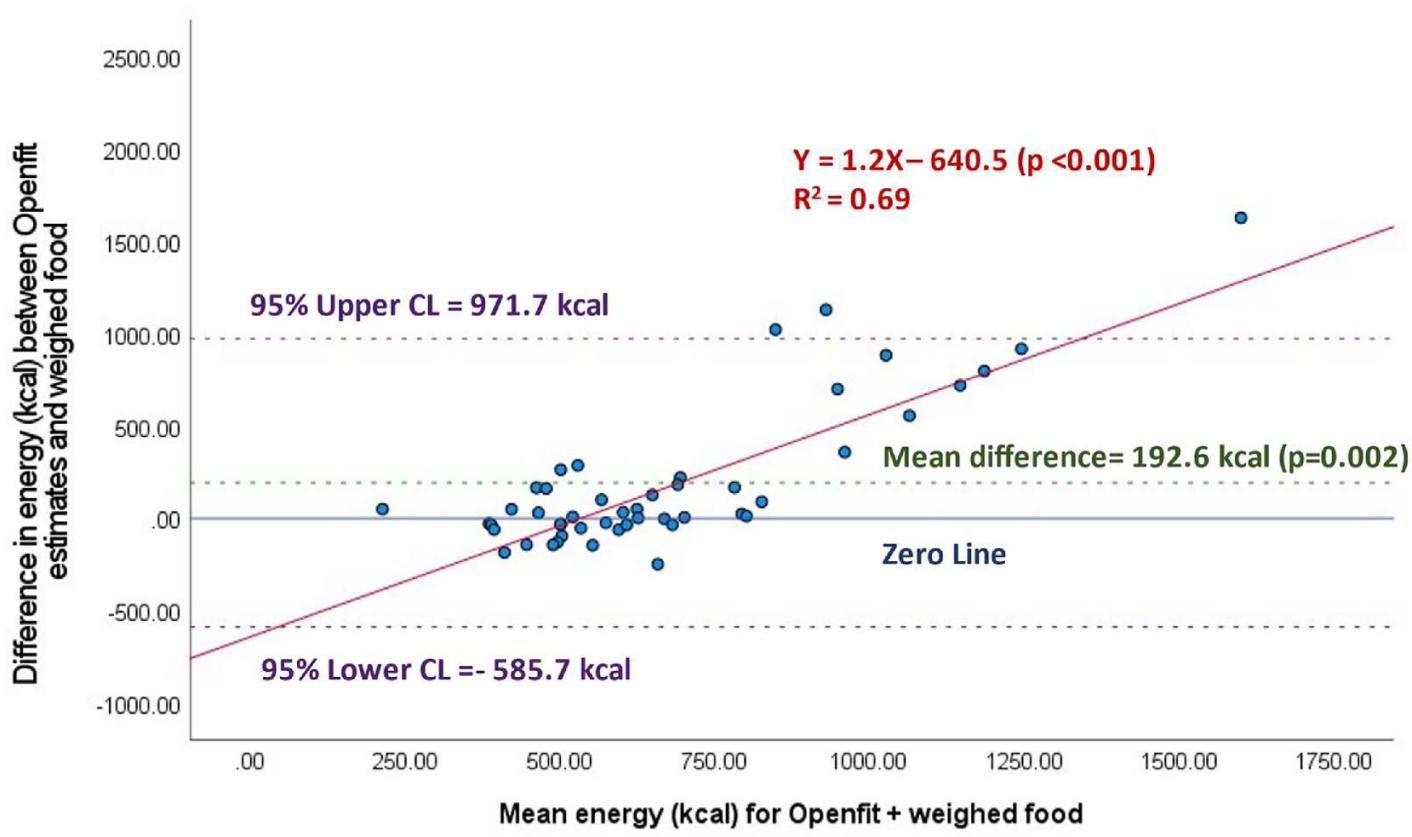
Semiautomated estimation (Nutrition AI estimates with user adjustment) of energy (kcal) per meal (*n* = 48), using the Openfit app.

Across all meals (*n* = 48) energy estimates were 577 ± 150 kcal for weighed meals, 826 ± 490 kcal for automated estimates, and 769 ± 445 kcal for semiautomated estimates. The difference between automated and semiautomated estimates of energy and weighed estimates were 249 ± 485 kcal (*P* = 0.001) (Figure 1), and 193 ± 397 kcal (*P* = 0.002) (Figure 2), respectively. Therefore, automated and semiautomated estimates of energy were 43% and 33% higher than weighed estimates, respectively. For automated and semiautomated energy estimates, the Bland–Altman plots demonstrate significant proportional bias, with higher energy meals contributing to the largest error in energy (both *P* values < 0.001) (FIGURE 1, FIGURE 2). The linear relationship between weighed and automated energy estimates from the Nutrition AI was not significant (*R*^2^ = 0.03, *P* = 0.09) (Supplementary Figure 6). Whereas there was a significant positive linear relationship between weighed and semiautomated energy estimates (Supplementary Figure 7) (*R*^2^ = 0.22, *P* < 0.001), and for every 1 calorie increase in meals provided, semiautomated energy estimates in Openfit increased by 1.4 calories.

**FIGURE 3:**
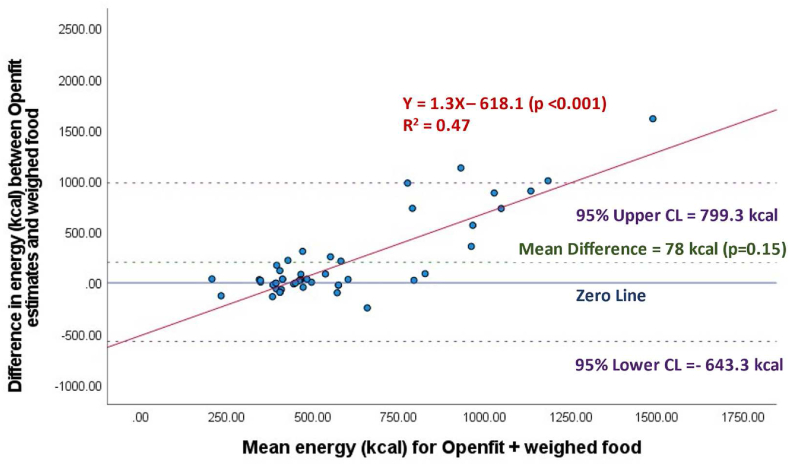
Automated estimation of energy (kcal) per meal (*n* = 48), by Nutrition AI in the Openfit app, excluding beverages.

**FIGURE 4:**
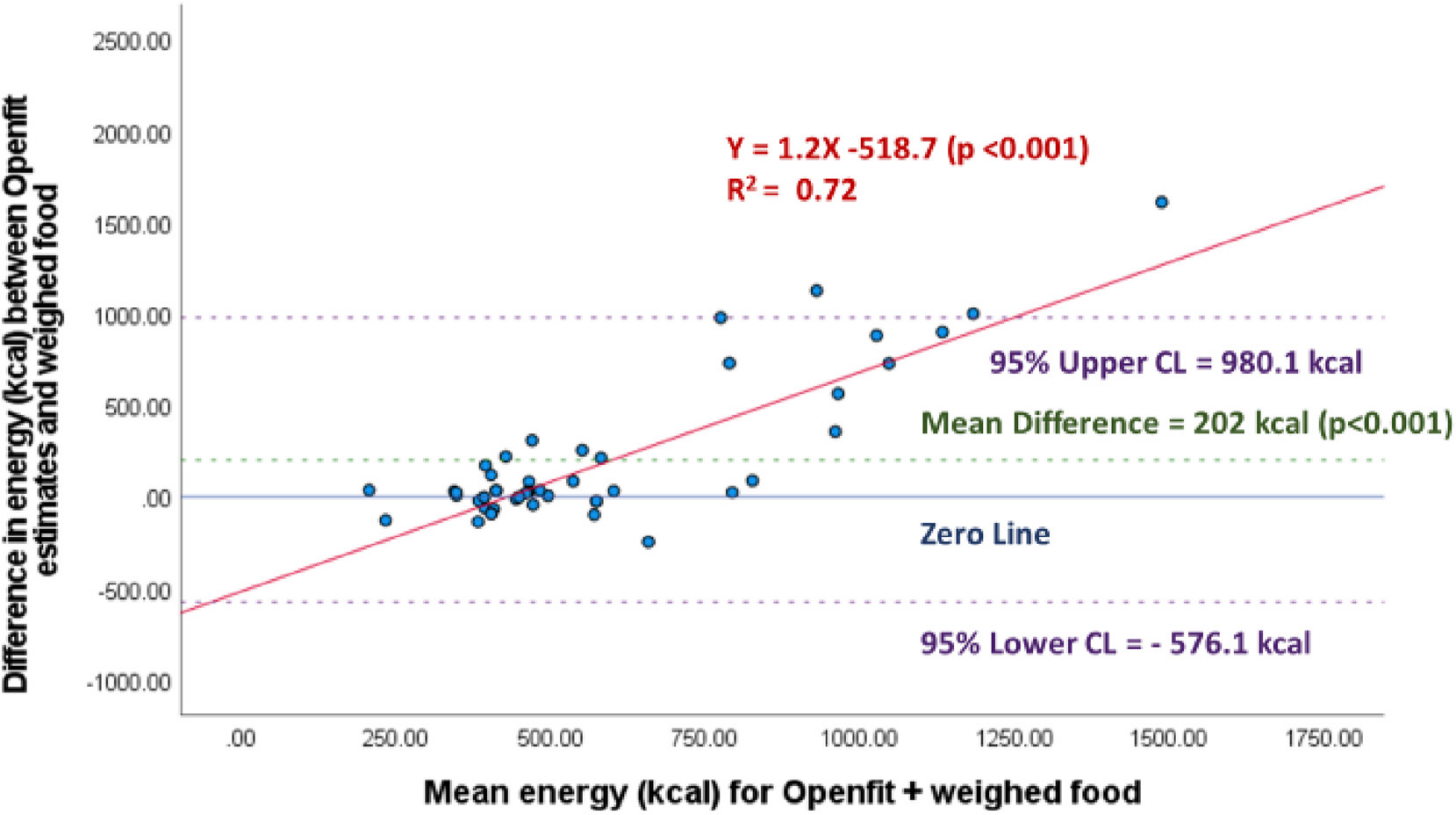
Semiautomated estimation (Nutrition AI estimates with user adjustment) of energy (kcal) per meal (*n* = 48), using the Openfit app, excluding beverages.

For the analyses excluding beverages, across all meals, energy estimates were 481 ± 148 kcal for weighed meals, 560 ± 343 kcal for automated estimates, and 683 ± 454 kcal for semiautomated estimates. The difference between automated and semiautomated estimates of energy and weighed estimates were 78 ± 368 kcal (*P* = 0.15) (Figure 3), and 202 ± 397 kcal (*P* < 0.001) (Figure 4), respectively. Therefore, automated, and semiautomated estimates of energy were 16% and 42% higher than weighed estimates, respectively. Excluding beverages from analyses, for automated (*R*^2^ = 0.47, *P* < 0.001) and semiautomated (*R*^2^ = 0.72, *P* < 0.001) energy estimates, the Bland–Altman plots demonstrated significant proportional bias, with higher energy meals contributing to the largest error in energy (FIGURE 3, FIGURE 4). Also, the linear relationship between weighed food and automated estimates were nonsignificant (*R*^2^ = 0.002, *P* = 0.79) (Supplementary Figure 8), and there was a significant linear relationship between weighed food and semiautomated estimates (*R*^2^ = 0.27, *P* < 0.001) (Supplementary Figure 9).

For automated estimation, at the meal level, error in energy (kcal) with weighed meals, ranged from 31% for the salad meal to 82% for the Chicken meal (Table 6). For semiautomated estimates of energy, the error with weighed estimates ranged from −5% for the Pork chop meal to 112% for the Chicken meal (Table 6). When excluding beverages from the analyses, error for automated estimates ranged from 7% for the Pork chop meal to 45% for the Burger meal, and error for semiautomated estimates ranged from 2% for the Pork chop meal to 128% for the Chicken meal (Table 7).

**TABLE 6:** Comparison of mean, percent error, ±10% and ±25% energy (kcal) for weighed (criterion method) food items compared with energy estimated using the Openfit application through automated (Nutrition AI) and semiautomated (Nutrition AI with user adjustment) methods

	*n*	Weighed kcal Mean ± SD	Automated kcal Mean ±SD	% Error Mean ± SD^1^	Semiautomated Mean ±SD	% Error Mean ±SD^2^	Automated Within ±10%, *n* (%)	Semiautomated Within ±10%, *n* (%)	Automated Within ±25%, *n* (%)	Semiautomated Within ±25%, *n* (%)
Chicken meal	9	605 ± 218	1160 ± 578	82 ± 34	1283 ± 637	112 ± 89	0 (0)	0 (0)	0 (0)	1 (11)
Burger meal	10	488 ± 91	641 ± 138	39 ± 53	509 ± 146	7 ± 35	3 (30)	3 (30)	5 (50)	5 (50)
Pizza meal	9	603 ± 199	742 ± 317	39 ± 96	845 ± 410	54 ± 102	3 (33)	3 (33)	5 (56)	5 (56)
Pork chop meal	10	554 ± 65	888 ± 511	63 ± 98	524 ± 78	−5 ± 14	0 (0)	7 (70)	4 (40)	9 (90)
Salad meal	10	629 ± 108	714 ± 636	31 ± 162	712 ± 272	13 ± 41	2 (20)	5 (50)	5 (50)	7 (70)

Food items marked as an omission were excluded, *n* = 5 items.

% Error = [(automated or semiautomated kcal – weighed kcal)/weighed kcal] ∗100.

1 Percent error between energy estimates for weighed food compared with automated energy estimates.

2 Percent error between energy estimates for weighed food compared with semiautomated energy estimates.

**TABLE 7:** Comparison of mean, percent error, ±10% and ±25% energy (kcal) for weighed (criterion method) food items compared with energy estimated using the Openfit application through automated (Nutrition AI) and semiautomated (Nutrition AI with user adjustment) methods, excluding beverages

	*n*	Weighed kcal Mean ± SD	Automated kcal Mean ± SD	% Error Mean ± SD^1^	Semiautomated Mean ± SD	% Error Mean ± SD^2^	Automated Within ±10%, *n* (%)	Semiautomated Within ±10%, *n* (%)	Automated Within ±25%, *n* (%)	Semisutomated Within ±25%, *n* (%)
Chicken meal	9	523 ± 190	556 ± 83	20 ± 43	1204 ± 645	128 ± 105	3 (33)	1 (11)	4 (44)	1 (11)
Burger meal	10	396 ± 70	551 ± 128	45 ± 54	436 ± 134	98 ± 43	4 (40)	2 (20)	4 (40)	5 (50)
Pizza meal	9	603 ± 199	645 ± 318	20 ± 91	828 ± 420	50 ± 103	3 (33)	4 (44)	4 (44)	6
Pork chop meal	10	423 ± 29	449 ± 125	7 ± 35	433 ± 38	2 ± 5	0 (0)	10 (100)	6 (60)	10 (100)
Salad meal	10	456 ± 82	596 ± 675	37 ± 162	543 ± 254	20 ± 60	1 (10)	3 (30)	5 (50)	7 (70)

Food items marked as an omission were excluded, *n* = 5 items.

% Error = [(automated or semiautomated kcal – weighed kcal)/weighed kcal] ∗100.

1 Percent error between energy estimates for weighed food compared with automated energy estimates.

2 Percent error between energy estimates for weighed food compared with semiautomated energy estimates.

Stratified by FNDDS food groups, for automated energy estimation, across all food items, error ranged from −28% for milk and milk products to 314% for sugars, sweets, and beverages (Table 8). For semiautomated energy estimation, error ranged from −10% for sugars, sweets, and beverages to 332% for grain products (Table 8). Supplementary Table 2 details misreporting of energy intake at the food item level by FNDDS food groups. On average, for most food items, automated and semiautomated estimates of energy intake were overestimated. The results for error in kilocalories within ±10% and ±25% weighed estimates in TABLE 6, TABLE 7, TABLE 8 detail the intervariability of energy estimates between participants.

**TABLE 8:** Comparison of mean, percent error, ±10% and ±25% energy (kcal) for weighed (criterion method) food items compared with energy estimated using the Openfit application through automated (Nutrition AI) and semiautomated (Nutrition AI with user adjustment) methods, by FNDDS food groups

	*n*	Weighed kcal Mean ± SD	Automated kcal Mean ± SD	% Error Mean ± SD^1^	Semiautomated Mean ± SD	% Error Mean ± SD^2^	Automated Within ± 10%, *n* (%)	Semiautomated Within ± 10%, *n* (%)	Automated Within ± 25%, *n* (%)	Semiautomated Within ± 25%, *n* (%)
Milk and milk products	12	160 ± 60	111 ± 56	−28 ± 32	163 ± 73	9 ± 45	0 (0)	2 (17)	5 (42)	6 (50)
Meat, poultry, fish and mixtures	37	199 ± 80	282 ± 316	60 ± 196	259 ± 140	42 ± 90	13 (35)	12 (32)	13 (35)	15 (41)
Grain products	39	170 ± 131	173 ± 124	78 ± 269	332 ± 379	102 ± 200	7 (18)	5 (13)	11 (28)	10 (26)
Fruits	19	86 ± 23	88 ± 29	6 ± 29	104 ± 25	23 ± 20	1 (5)	1 (5)	5 (26)	7 (37)
Vegetables	73	27 ± 19	53 ± 71	251 ± 621	25 ± 23	65 ± 118	8 (11)	14 (19)	9 (12)	20 (27)
Fats, oils, and salad dressings	38	140 ± 54	105 ± 201	−22 ± 116	145 ± 198	2 ± 103	6 (16)	15 (39)	6 (16)	22 (58)
Sugars, sweets, and beverages	37	78 ± 58	314 ± 434	255 ± 420	66 ± 59	−10 ± 50	0 (0)	14 (38)	2 (5)	19 (51)

Food items marked as an omission were excluded, *n* = 5 items.

% Error = [(automated or semiautomated kcal – weighed kcal)/weighed kca]) ∗100.

1 Percent error between energy estimates for weighed food compared with automated energy estimates.

2 Percent error between energy estimates for weighed food compared with semiautomated energy estimates.

### CSUQ and USS results

With 1 being the highest usability and 7 being the lowest, across all participants, the mean usability score for the CSUQ for Openfit was 2.4 (± 0.22). For the USS, with 1 being the lowest and 6 being the most favorable score, means scores ranged from 4.1 (± 1.3) for the question “How satisfied are you with the application for estimating the amount of food provided?” to 5.5 ± 0.7 for the question “How much did the training help prepare you for using the app?” (Table 9).

**TABLE 9:** User Satisfaction Survey results (*n* = 24)

	Score Mean ± SD
How **satisfied** are you with the application for identifying the food provided?	4.1 ± 1.3
How **satisfied** are you with the application for estimating the amount of food provided?	4.0 ± 1.5
How **easy** was it to use the application to identify the food provided?	4.2 ± 1.4
How **easy** was it to use the application for estimating the amount of food provided?	4.0 ± 1.6
How much did the training help prepare you for using the app?	5.5 ± 0.7

For the open-ended responses in the USS, key themes emerged. For the question, “*which aspects of Openfit did you find the easiest to use for recording the food provided,*” key themes included Nutrition AI, manual recording, and the overall app. Across all participants, 79% (19/24), 29% (7/24), and 12% (3/24) commented that the Nutrition AI, manual recording, and the overall app, were the easiest to use, respectively. For the question, “*Which aspects of Openfit did you find the most difficult to use for recording the food provided*,” key themes also centered around the Nutrition AI, manual recording, and the overall app. Across all participants, 54% (13/24), 62% (15/24), and 29% (7/24) of participants reported that the Nutrition AI, manual recording, and the overall application were the most difficult to use, respectively. For the question, *please write any additional comments you have about the pros and cons of using the Openfit app*, 4 key themes emerged. For the pros, 1 key theme was apparent, with 12% (3/24) of participants commenting on the pros of the Nutrition AI. For the cons, 3 key themes were evident with 42% (10/24), 29% (7/24), and 21% (5/24) of participants commenting on the cons of the Nutrition AI, manual recording, and overall app, respectively.

## Discussion

To our knowledge, this is the first study to evaluate the validity of an artificial intelligence-based mobile application to identify and quantify energy intake through short video capture without a physical reference marker, using the Openfit app. Errors at the meal level, analyses excluding beverages, and analyses by FNDDS food groups coupled with participants’ feedback on the Openfit app, highlight the strengths and areas for future development of Nutrition AI in dietary assessment applications.

Overall, for automated identification, 46% of food items logged into Openfit were an exact match, and 13% were intrusions. These results varied from 27% exact matches for the pizza meal to 67% for the hamburger meal. Therefore, there was large variability in the percentage of exact matches depending on the meal served. Both exact matches and intrusions significantly improved with user correction (semiautomated methods). For semiautomated methods, exact matches ranged from 81% for the salad meal to 98% for the hamburger meal, and there were no intrusions. This demonstrates the importance of allowing users to manually change food labels automatically identified through Nutrition AI. Using a traditional paper-based method, previous research reported that adolescents in the United States could correctly identify 79%–85%of food items provided [27]. Therefore, the accuracy of semiautomated identification for the current study was relatively high.

After removing beverages from analyses, exact matches for automatic identification improved to 57%. For semiautomated identification, exact matches improved slightly to 90% after beverages were removed. Improving Nutrition AI to automatically identify beverages appears to be a priority.

For results stratified by FNDDS food groups, for automated identification, exact matches were lowest for the sugars, sweets, and beverages group, the fats, oils, and salad dressings group, and the milk and milk products group, with exact matches being 0%, 8%, and 17%, respectively. For semiautomated identification exact matches for these food groups significantly improved to 81%, 92%, and 50%, respectively. These results highlight that improvements to Nutrition AI could be focused on specific food groups, users may need additional training for logging specific food groups, and again demonstrate the importance of users being able to manually change the identification of food items. These results are consistent with previous studies reporting that automated identification is challenging for amorphous foods (eg, salad dressing) and for food items that look similar but can have different nutrient profiles (eg, Diet Coke compared with Coca Cola) [2], particularly if no container, wrapper, or logo is included in the product being identified.

Application features and methods for validity studies differ considerably between dietary assessment applications, making it challenging to compare results between studies. For example, for automated identification, a combination of AI models [[28], [29], [30]], handcrafted features (shape, color, texture, and location), and deep visual features are used in applications for food identification [2]. For manual methods of food identification, dietary assessment applications differ in the food search function, food portion size options, and nutrient database used. Studies assessing the validity of mobile applications for food identification may be laboratory-based studies, or community-dwelling studies, and may assess validity at the item, meal, or day level. In addition, what constitutes an “exact match” for food identification differs between studies. Despite these differences in methodologies, previous studies have reported that accurate identification via automated estimates varies widely from 44% to 97.2% [[31], [32], [33], [34], [35], [36], [37], [38], [39]]. These tests are often completed under ideal conditions (eg, 1 item per plate, food served on a plain white plate, well-photographed meal, in a well-lit room) [32,34] and using a limited food data set created for training the AI [[33], [34], [35],^37^,^38^]; therefore, they do not reflect results in a real-life setting. In the current study, 46% of food items logged into Openfit were an exact match; therefore, the accuracy of the Nutrition AI in Openfit for automatic identification was in the expected range. However, consistent with the current literature, the accuracy of the Nutrition AI in Openfit widely differed depending on the type of meal item being tested (eg, accuracy was lower for amorphous foods).

On average, with 6 being the most favorable score, participants rated 4.1 (± 1.3) for the question “how satisfied are you with the application for identifying the food provided?,” and 4.2 (±1.4) for the question “how easy was it to use the application to identify the food provided?” These scores reflect that improvements are needed to improve the usability of Openfit for identifying food items. Participants suggested improvements to the Nutrition AI, manual recording, and overall application for food identification.

Across all meals, error was 43% and 33% for automated and semiautomated energy estimates, respectively. For automated energy estimates, the error ranged from 31% for the salad meal to 82% for the Chicken meal. For semiautomated energy estimates errors ranged from −5% for the Pork chop meal to 112% for the Chicken meal. Therefore, for both automated and semiautomated energy estimates, there was a large variability in error depending on the type of meal being tested. Within meals, variability in estimates also existed. For example, for the pizza meal, 50% of participants had automated and semiautomated estimates within ±25%. Further research is needed, on a larger sample, to determine participant characteristics that may influence error in automated and semiautomated energy estimates.

After removing all beverages from analyses, the error in automated estimates was only 16%, and error for semiautomated estimates was 42%. Therefore, a large amount of error in automated energy estimates was driven by beverages. The analyses by FNDDS food groups support these results with a 255% error found for automated energy estimates for the sugars, sweets, and beverages group. For automated energy estimates, a large amount of error was also driven by the vegetables group, which had a 251% error. For semiautomated energy estimates, the FNDDS stratified analyses detail that grain products were contributing to the largest amount of error (332% error), and error for the Sugar, sweets, and beverages was low (−10% error). Because the error for Sugar, sweets, and beverages was already low for semiautomated estimates, this explains why removing beverages from analyses did not improve the overall error. Within the grain products group, wild rice had the largest error for semiautomated estimates, with a 406% error (Supplementary Table 2). This error was unique to semiautomated estimates, as the error for automated energy estimates for wild rice was 23% (Supplementary Table 2). Therefore, for analyses excluding beverages, regardless of participants identifying wild rice correctly, this large error in semiautomated energy estimation for wild rice contributed to overall semiautomated energy estimates being higher than overall automated energy estimates. Also, with or without beverages, the Bland–Altman plots demonstrated that higher energy meals had the largest error. Therefore, for improving automated and semiautomated energy estimates with Openfit, focus should be given to improving estimates for specific food groups with the largest error, and to ensuring that users can accurately use the Nutrition AI to estimate energy intake across meals of varying energy levels.

Overall, 41% of food items were a far match for automated identification, and 23% were a far match for semiautomated identification. Some far matches are close in nutrient composition, e.g., wild rice compared with brown rice, whereas other far matches differ substantially in nutrient composition, e.g., wild rice compared with pizza. Therefore, to improve the validity of energy estimates through Nutrition AI, ideally most food items should be an exact match. In terms of automated identification, improvements to the machine learning mechanisms are needed to minimize the occurrence of far matches. In this study, participants engaged with simulated meals, which could have increased the prevalence of far matches. If participants had the opportunity to order, prepare, or consume the test meals, it is plausible that the occurrence of far matches might have been lower. However, further investigation is required to ascertain these potential effects.

Previous studies have reported underreporting of energy intake from manual logging of ≤30% [2,[40], [41], [42], [43], [44]]. Errors from image-based dietary assessment methods, with a fiduciary marker, have also been reported as comparable to errors from traditional dietary assessment methods [45]. These image-based studies were between 1 and 7 days in length and conducted in a community-dwelling setting [45]. In the current laboratory-based study, error in semiautomated energy estimates was 33% error, which is similar to these previous studies. However, unlike the community-based studies, energy intake was overestimated with Openfit. A pilot study assessing the validity of the PortionSize app, which uses embedded food templates and a fiduciary marker to estimate portion size, had a study design more comparable to the current study [11]. Despite the technologies being different, both the PortionSize study and the current pilot were validity studies conducted in a laboratory-based setting with simulated meal provision [11]. The PortionSize application overestimated energy intake by 12.7% at the meal level [11]. It is important to conduct further validity testing of Nutrition AI within dietary assessment applications to assess if error in energy estimates differs in a community-dwelling environment.

Once beverages were excluded from analyses, error from automated energy estimates was only 16%, which is relatively low compared with previous studies [45]. This error is especially low given no fiducial marker is required for estimating food volume with Openfit. However, for semiautomated estimates, without beverages, error was 42%, which is relatively high. This demonstrates that for accurate energy estimation additional tools may be needed to assist Openfit users with semiautomated estimates of energy intake. Possible tools include using a scale to weigh food items that are the most difficult to manually quantify, or using visual guides for portion size estimation, for example, the portion size guides used in ASA-24 [46].

Although only a small number of foods (2%) were marked as omissions, 2/5 of the omissions were due to there being no nutrient information for pizza in the Openfit database. This highlights that the overreporting of energy intake in the current study may be due to participant error or errors in the nutrient database. Unfortunately, there is no published information on the data sets used to create the Openfit nutrient database; therefore, it is difficult to determine the exact cause of these errors. This demonstrates the importance of monitoring the accuracy of nutrient databases used for commercially based dietary assessment applications.

On average, with 6 being the best score, participants rated 4.0 (±1.5) for the question “how satisfied are you with the application for estimating the amount of food provided?,” and 4.0 (±1.6) for the question "How easy was it to use the application for estimating the amount of food provided?.” Similar to participant feedback on identification of food items, participants suggested improvements to the Nutrition AI, manual estimation, and overall application for improving the usability of Openfit for estimating energy intake.

One of the strengths of the present study lies in the discreet weighing of meals by the PBRC Metabolic Kitchen. Notably, the energy content of these meals was concealed from all other study staff and participants, ensuring a blind approach. Furthermore, the study's robustness is reinforced by the randomization of participants to 1 of 5 study menus. To examine potential proportional bias resulting from serving size, the meals were deliberately varied in energy content, providing additional layers of strength.

Limitations of the study include most participants were female, a healthy body weight and had some college education or greater; therefore, the study sample was not representative of the wider US population. The study was conducted in a laboratory-based setting, so a larger study with more food and in a community-dwelling environment is needed. Also, the Nutrition AI in Openfit may perform better with packaged food because the user can scan barcodes or use the nutrition facts panel to help identify and quantify foods, and we did not include packaged foods in the current study. The automated and semiautomated energy estimates in the Openfit application were generated from the nutrient database used by Openfit; however, there is no published information on this nutrient database. Finally, we only assessed the validity of the Nutrition AI on food provided. To examine the accuracy of the Nutrition AI more thoroughly for recording food intake data, future research should assess the validity of the Nutrition AI on both food served and food waste. Unfortunately, because the completion of data collection for the current study, Openfit is no longer available. Therefore, further research of the validity of Nutrition AI for dietary assessment would need to be conducted on an application with similar technology, such as MyFitnessPal, the Simple app, or Naiya. Nutrition AI is constantly evolving, for example, the Naiya application includes a virtual scale for volume estimation of food [47], which needs to be accounted for when comparing results of future validation studies across these commercially available mobile applications. However, the results of the current study are still valuable, and can serve as a framework for future studies analyzing the validity of artificially intelligence-based dietary assessment applications.

In conclusion, the accuracy of automated identification of food items with the Nutrition AI in the Openfit application was comparable to previous image-based dietary assessment technologies. The validity of semiautomated identification of foods with Openfit was relatively high, and exact matches were statistically better than for automated identification. This demonstrates the importance of users being able to manually change food items automatically identified through Nutrition AI. Error for automated energy estimates with the Nutrition AI in Openfit was relatively high; however, most error was driven by the sugars, sweets, and beverages FNDDS group. Once beverages were removed from analyses, automated energy estimates were not statistically different to weighed estimates. Error in semiautomated energy estimates was comparable to traditional dietary assessment methods, and most error was driven by the grain products FNDDS group. Participants suggested improvements to the Nutrition AI, manual estimation, and overall application for improving the usability of Openfit for identifying food items and estimating energy intake, which may improve overall usability scores.

## Author contributions

The authors’ responsibilities were as follows – CPL, CKM, JWA: formulated the research question(s); CPL, CKM, JWA: designed the research; CPL: conducted the research; CPL, ENC: analyzed the data; and all authors: interpreted the findings; and all authors: read and approved the final manuscript.

## Conflict of interest

All authors declare no conflicts of interest.

## Funding

This work was supported by the National Institute of Diabetes and Digestive and Kidney Diseases grants R01 DK124558, P30 DK072476, and T32 DK064584); Louisiana Clinical and Translational Science Center grant U54GM104940; and the United States Department of Agriculture (USDA) National Institute of Food and Agriculture (NIFA) grant 2023-67012-39409.

## Data availability

Data described in the manuscript, code book, and analytic code will be made available from the corresponding author, CPL, upon reasonable request.

## Declaration of interests

The authors declare that they have no known competing financial interests or personal relationships that could have appeared to influence the work reported in this paper.

## Appendix A Supplementary data

Multimedia component1 Multimedia component1

Multimedia component2 Multimedia component2
