# Supplementary material for: Validity of an Artificial Intelligence-Based Application to Identify Foods and Estimate Energy Intake Among Adults: A Pilot Study
Source: Curr Dev Nutr. 2023 Sep 29;7(11):102009. doi: 10.1016/j.cdnut.2023.102009 (PMC10656219; doi:10.1016/j.cdnut.2023.102009)

Supplementary Materials

| Supplementary Table 1. Frequency of food items correctly identified through automated (Nutrition AI) and semi-automated (Nutrition AI with user adjustment) methods, sorted by FNDDS food groups. | | | | | | | |
| --- | --- | --- | --- | --- | --- | --- | --- |
|  |  | Automated Identification | | | Semi-Automated Identification | | |
|  | n | Exact^1^ | Far^2^ | Intrusion^3^ | Exact | Far | Intrusion |
| Milk and Milk Products |  |  |  |  |  |  |  |
| Hamburger Meal-cheese | 3 | 2 | 0 | 1 | 3 ↑ | 0 ↔ | 0 ↓ |
| 1% milk | 9 | 0 | 8 | 1 | 3 ↑ | 6 ↓ | 0 ↓ |
| Meat, Poultry, Fish and Mixtures |  |  |  |  |  |  |  |
| Chicken Meal-chicken breast | 9 | 4 | 4 | 1 | 8 ↑ | 1 ↓ | 0 ↓ |
| Hamburger (mixed dish) | 6 | 6 | 0 | 0 | 6 ↔ | 0 ↔ | 0 ↔ |
| Hamburger Meal-meat patty | 3 | 2 | 0 | 1 | 3 ↑ | 0 ↔ | 0 ↓ |
| Pork chop | 10 | 0 | 10 | 0 | 5 ↑ | 5 ↓ | 0 ↔ |
| Salad Meal-chicken breast | 9 | 7 | 1 | 1 | 9 ↑ | 0 ↓ | 0 ↓ |
| Grain Products |  |  |  |  |  |  |  |
| Wild rice | 9 | 9 | 0 | 0 | 9 ↔ | 0 ↔ | 0 ↔ |
| Hamburger Meal-hamburger bun | 3 | 0 | 2 | 1 | 3 ↑ | 0 ↓ | 0 ↓ |
| Chocolate chip cookie | 9 | 8 | 1 | 0 | 9 ↑ | 0 ↓ | 0 ↔ |
| Pizza | 8 | 0 | 8 | 0 | 7 ↑ | 1 ↓ | 0 ↔ |
| Oreo cookie | 10 | 10 | 0 | 0 | 10 ↔ | 0 ↔ | 0 ↔ |
| Fruits |  |  |  |  |  |  |  |
| Orange | 9 | 7 | 2 | 0 | 7 ↔ | 2 ↔ | 0 ↔ |
| Apple | 10 | 10 | 0 | 0 | 10 ↔ | 0 ↔ | 0 ↔ |
| Vegetables |  |  |  |  |  |  |  |
| Chicken Meal- carrot | 8 | 8 | 0 | 0 | 8 ↔ | 0 ↔ | 0 ↔ |
| Hamburger Meal-tomato | 3 | 3 | 0 | 0 | 3 ↔ | 0 ↔ | 0 ↔ |
| Hamburger Meal-onions | 3 | 3 | 0 | 0 | 3 ↔ | 0 ↔ | 0 ↔ |
| Ketchup | 9 | 8 | 0 | 1 | 9 ↑ | 0 ↔ | 0 ↓ |
| Salad Meal (mixed dish) | 9 | 2 | 3 | 4 | 5 ↑ | 4 ↓ | 0 ↔ |
| Salad Meal-lettuce | 1 | 0 | 1 | 0 | 0 ↔ | 1 ↔ | 0 ↔ |
| Salad Meal-tomato | 1 | 1 | 0 | 0 | 1 ↔ | 0 ↔ | 0 ↔ |
| Broccoli | 10 | 9 | 1 | 0 | 9 ↔ | 1 ↔ | 0 ↔ |
| Salad Meal-tomato | 9 | 6 | 3 | 0 | 8 ↑ | 1 ↓ | 0 ↔ |
| Salad Meal-carrot | 10 | 10 | 0 | 0 | 10 ↔ | 0 ↔ | 0 ↔ |
| Salad Meal-lettuce | 9 | 0 | 6 | 3 | 8 ↑ | 1 ↓ | 0 ↓ |
| Salad Meal-tomato and lettuce | 1 | 0 | 1 | 0 | 0 ↔ | 1 ↔ | 0 ↔ |
| Fats, Oils, and Salad Dressings |  |  |  |  |  |  |  |
| Chicken Meal- hard butter | 8 | 0 | 2 | 6 | 8 ↑ | 0 ↓ | 0 ↓ |
| Ranch dressing | 10 | 0 | 8 | 2 | 9 ↑ | 1 ↓ | 0 ↓ |
| Pork Meal- hard butter | 10 | 3 | 1 | 6 | 10 ↑ | 0 ↓ | 0 ↔ |
| Honey mustard dressing | 10 | 0 | 10 | 0 | 8 ↑ | 2 ↓ | 0 ↔ |
| Sugars, Sweets, and Beverages |  |  |  |  |  |  |  |
| Diet Coke | 10 | 0 | 9 | 1 | 8 ↑ | 2 ↓ | 0 ↓ |
| Coca-Cola | 9 | 0 | 8 | 1 | 8 ↑ | 1 ↓ | 0 ↓ |
| Chicken Meal- sweet tea | 9 | 0 | 9 | 0 | 7 ↑ | 2 ↓ | 0 ↔ |
| Pork Meal- sweet tea | 9 | 0 | 7 | 2 | 7 ↑ | 2 ↓ | 0 ↓ |
| All data presented as n. ^1^Exact match (all eight digits match), ^2^Far match (first digit matches), and ^3^no match with the Food and Nutrient Database for Dietary Studies (FNDDS) food code. Food item marked as an omission and excluded, n = 5 items. ↑ frequency increased between automated and semi-automated estimates; ↓ frequency decreased between automated and semi-automated estimates; ↔ frequency for automated and semi-automate estimates were the same. | | | | | | | |

| Supplementary Table 2. Comparison of mean (±SD), ±10%, and ±25% energy (kcal) for weighed (criterion method) food items vs. energy estimated using the Openfit app through automated (Nutrition AI) and semi-automated (Nutrition AI with user adjustment) methods, sorted by FNDDS food groups. | | | | | | | | |
| --- | --- | --- | --- | --- | --- | --- | --- | --- |
|  |  | Weighed | Automated Estimation | | | Semi-Automated Estimation | | |
|  | n | Kcal  Mean (±SD) | Kcal  Mean (±SD) | Within ±10%  n (%) | Within ±25%  n (%) | Kcal  Mean (±SD) | Within ±10%  n (%) | Within ±25%  n (%) |
| Milk and Milk Products |  |  |  |  |  |  |  |  |
| Hamburger Meal-cheese | 3 | 63 (15) | 50 (35) | 0 | 1 | 85 (26) | 0↔ | 1↔ |
| 1% milk | 9 | 192 (15) | 132 (47) | 0 | 4 | 189 (65) | 2↑ | 5↑ |
| Meat, Poultry, Fish and Mixtures |  |  |  |  |  |  |  |  |
| Chicken Meal-chicken breast | 9 | 180 (47) | 237 (73) | 0 | 0 | 255 (150) | 0↔ | 0↔ |
| Hamburger (mixed dish) | 6 | 344 (45) | 281 (1) | 2 | 3 | 281 (1) | 2↔ | 3↔ |
| Hamburger Meal-meat patty | 3 | 117 (34) | 272 (158) | 1 | 1 | 267 (91) | 0↓ | 0↓ |
| Pork chop | 10 | 154 (1) | 222 (116) | 6 | 6 | 158 (19) | 7↑ | 9↑ |
| Salad Meal-chicken breast | 9 | 199 (63) | 421 (673) | 0 | 3 | 363 (209) | 3↑ | 3↔ |
| Grain Products |  |  |  |  |  |  |  |  |
| Wild rice | 9 | 138 (52) | 170 (0) | 5 | 5 | 699 (516) | 0↓ | 0↓ |
| Hamburger Meal-hamburger bun | 3 | 113 (28) | 25 (30) | 0 | 0 | 123 (0) | 0↔ | 2↑ |
| Chocolate chip cookie | 9 | 47 (15) | 193 (121) | 0 | 0 | 50 (20) | 4↑ | 4↑ |
| Pizza | 8 | 407 (10) | 337 (102) | 2 | 6 | 511 (329) | 1↓ | 4↓ |
| Oreo cookie | 10 | 137 (29) | 70 (0) | 0 | 0 | 175 (37) | 0↔ | 0↔ |
| Fruits |  |  |  |  |  |  |  |  |
| Orange | 9 | 68 (20) | 60 (5) | 0 | 0 | 90 (29) | 0↔ | 2↑ |
| Apple | 10 | 102 (9) | 114 (15) | 1 | 5 | 116 (13) | 1↔ | 5↔ |
| Vegetables |  |  |  |  |  |  |  |  |
| Chicken Meal- carrot | 8 | 44 (14) | 50 (9) | 6 | 6 | 55 (11) | 4↓ | 4↓ |
| Hamburger Meal-tomato | 3 | 7 (0) | 32 (1) | 0 | 0 | 22 (9) | 0↔ | 0↔ |
| Hamburger Meal-onions | 3 | 11 (0) | 24 (0) | 0 | 0 | 31 (15) | 0↔ | 0↔ |
| Ketchup | 9 | 16 (1) | 36 (5) | 0 | 0 | 23 (9) | 4↑ | 5↑ |
| Salad Meal (mixed dish) | 9 | 13 (3) | 130 (131) | 0 | 0 | 36 (24) | 1↑ | 1↑ |
| Salad Meal-lettuce | 1 | 5 (0) | 5 (0) | 1 | 1 | 27 (0) | 1↔ | 1↔ |
| Salad Meal-tomato | 1 | 6 (0) | 32 (0) | 0 | 0 | 16 (0) | 0↔ | 0↔ |
| Broccoli | 10 | 52 (14) | 28 (1) | 0 | 0 | 51 (22) | 0↔ | 2↑ |
| Salad Meal-tomato | 9 | 14 (1) | 33 (8) | 0 | 0 | 24 (10) | 1↑ | 1↑ |
| Salad Meal-carrot | 10 | 49 (4) | 30 (0) | 0 | 0 | 37 (12) | 1↑ | 2↑ |
| Salad Meal-lettuce | 9 | 13 (1) | 94 (127) | 0 | 1 | 18 (10) | 3↑ | 4↑ |
| Salad Meal-tomato and lettuce | 1 | 30 (0) | 28 (0) | 1 | 1 | 138 (0) | 0↓ | 0↓ |
| Fats, Oils, and Salad Dressings |  |  |  |  |  |  |  |  |
| Chicken Meal- hard butter | 8 | 112 (44) | 50 (17) | 1 | 1 | 126 (71) | 4↑ | 5↑ |
| Ranch dressing | 10 | 128 (62) | 184 (383) | 5 | 5 | 210 (378) | 0↓ | 6↑ |
| Pork Meal- hard butter | 10 | 115 (23) | 86 (94) | 0 | 0 | 108 (26) | 9↑ | 9↑ |
| Honey mustard dressing | 10 | 201 (16) | 90 (0) | 0 | 0 | 132 (82) | 2↑ | 2↑ |
| Sugars, Sweets, and Beverages |  |  |  |  |  |  |  |  |
| Diet Coke | 10 | 0 (0) | 97 (50) | 0 | 0 | 16 (47) | 8↑ | 8↑ |
| Coca-Cola | 9 | 92 (21) | 91 (54) | 0 | 2 | 73 (48) | 3↑ | 3↑ |
| Chicken Meal- sweet tea | 9 | 82 (28) | 604 (547) | 0 | 0 | 79 (40) | 2↑ | 3↑ |
| Pork Meal- sweet tea | 9 | 146 (28) | 487 (548) | 0 | 0 | 101 (67) | 1↑ | 5↑ |
| Food item marked as an omission and excluded, n = 5 items. ↑ frequency increased between automated and semi-automated estimates; ↓ frequency decreased between automated and semi-automated estimates; ↔ frequency for automated and semi-automate estimates were the same. *FNDDS: Food and Nutrient Database for Dietary Studies.* | | | | | | | | |

Supplementary Figure 1. Menu 1: Chicken Meal (400 kcal) and Hamburger Meal (400 kcal)


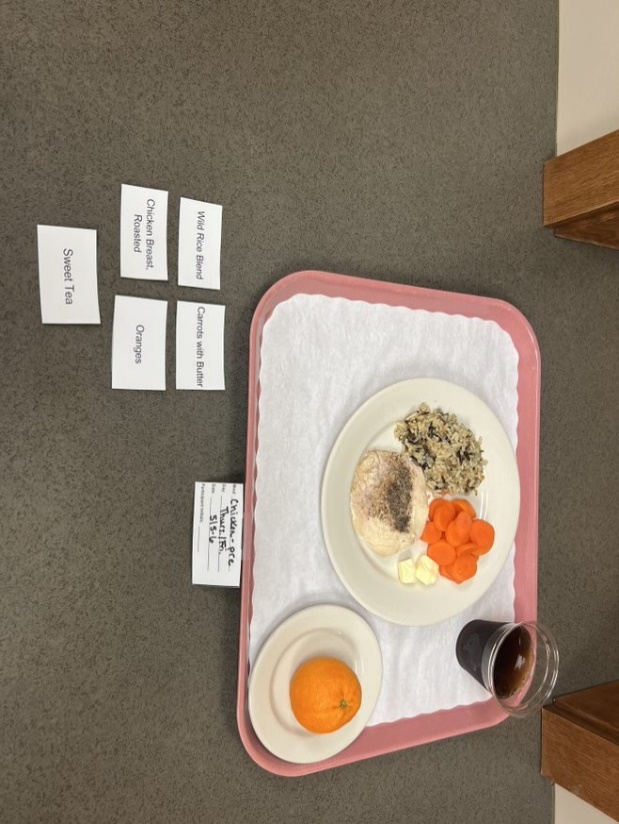


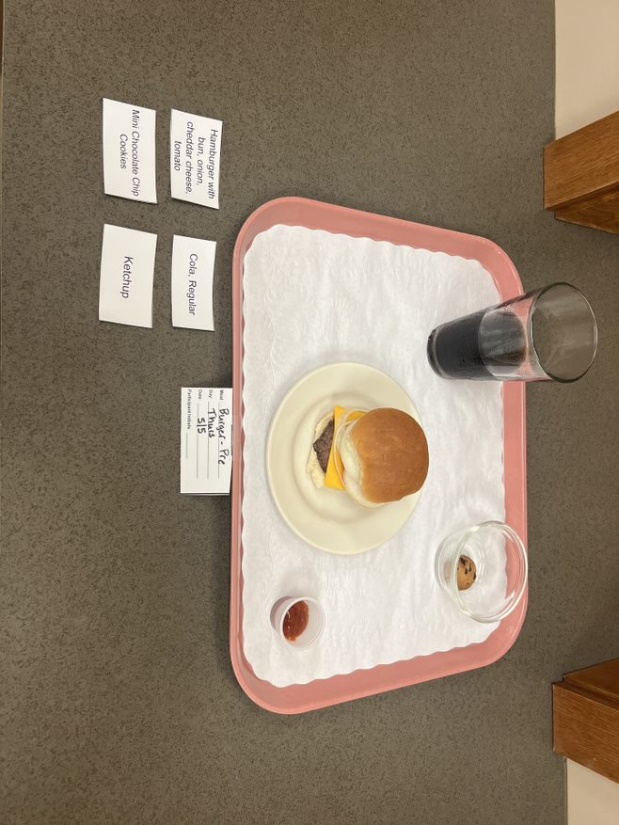


Supplementary Figure 2. Menu 2: Pizza Meal (800 kcal) and Chicken Meal (800 kcal)


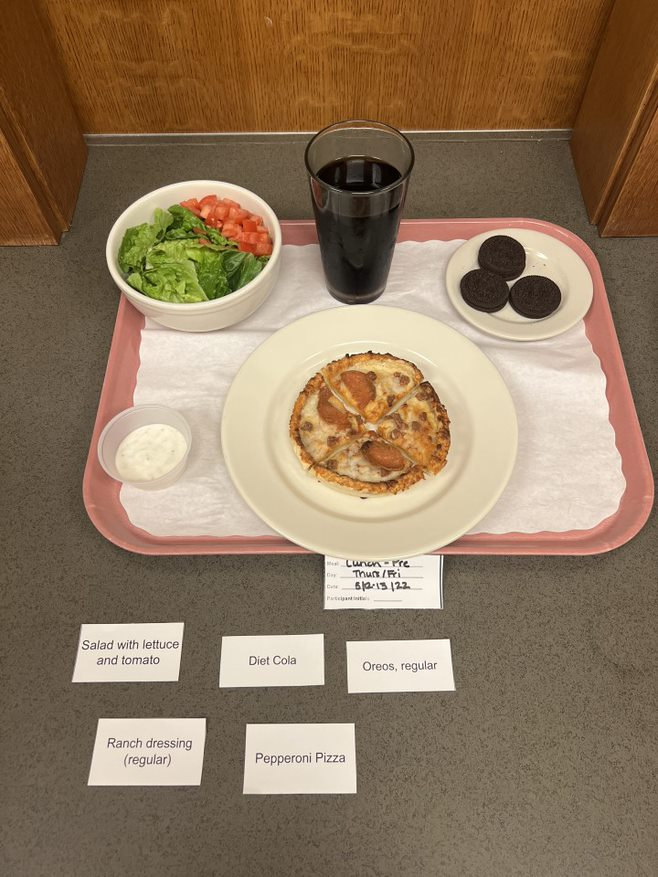


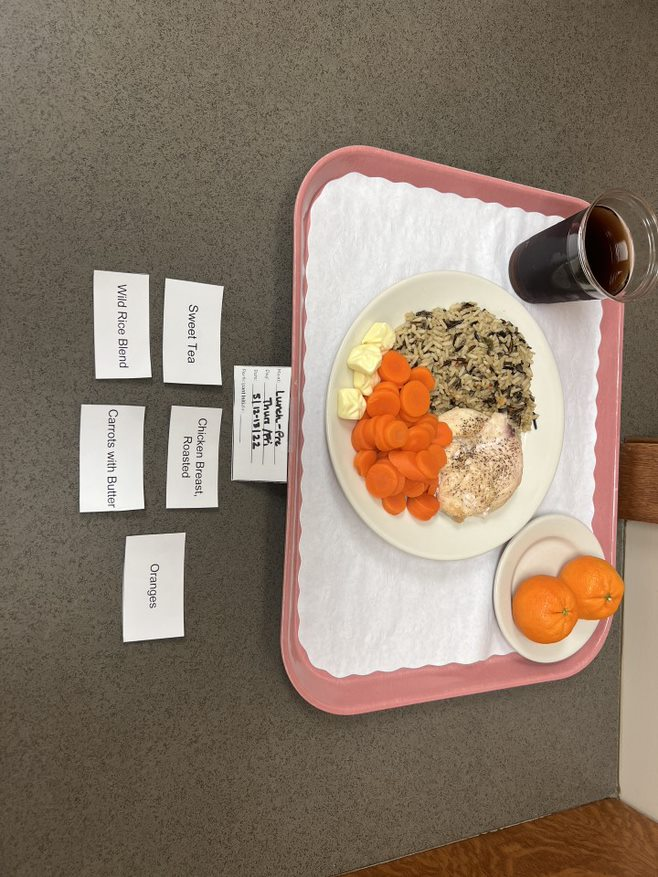


Supplementary Figure 3. Menu 3: Pizza Meal (500 kcal) and Pork Chop Meal (500kcal)


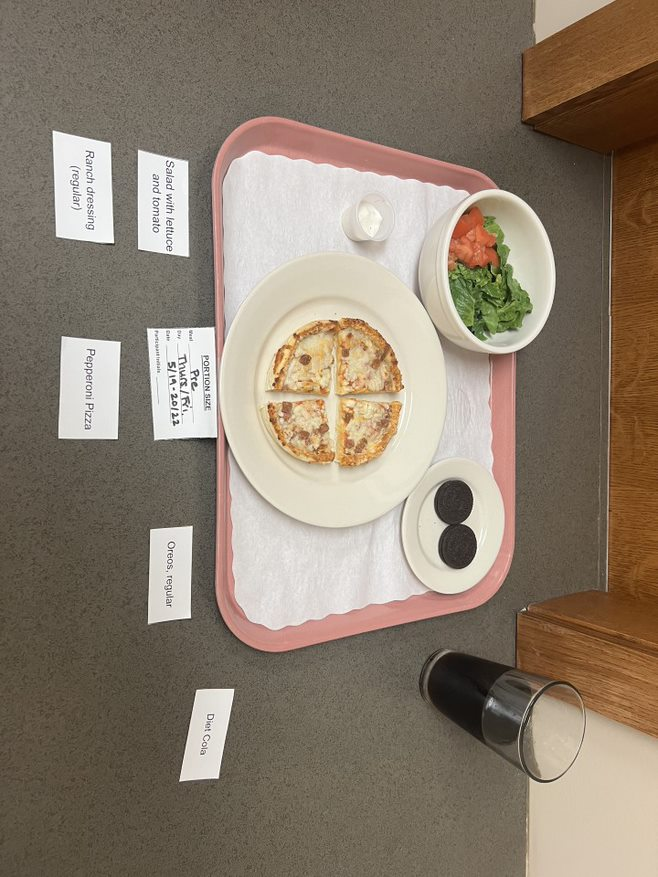


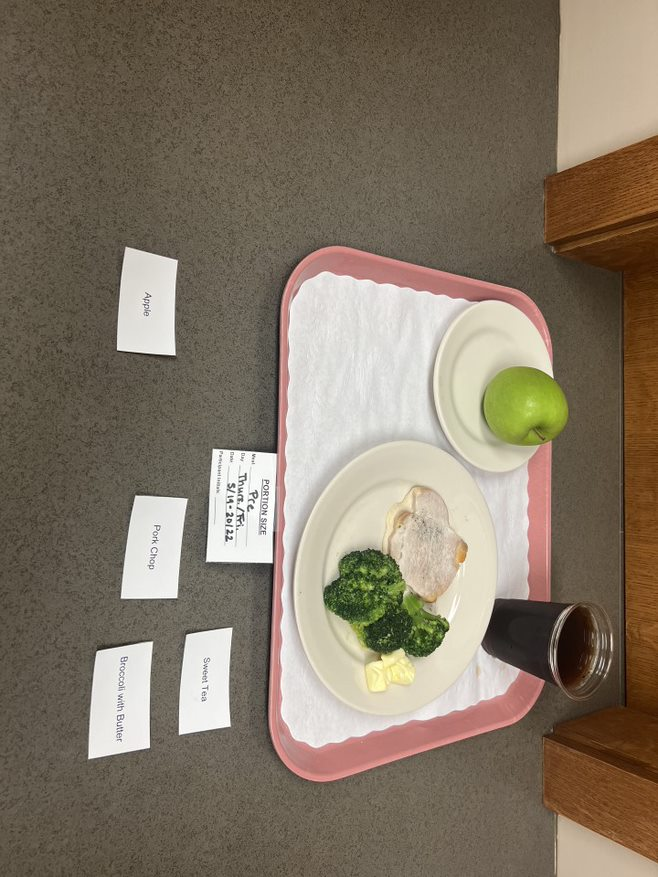


Supplementary Figure 4. Menu 4: Pork Chop Meal (700 kcal) and Salad Meal (700 kcal)


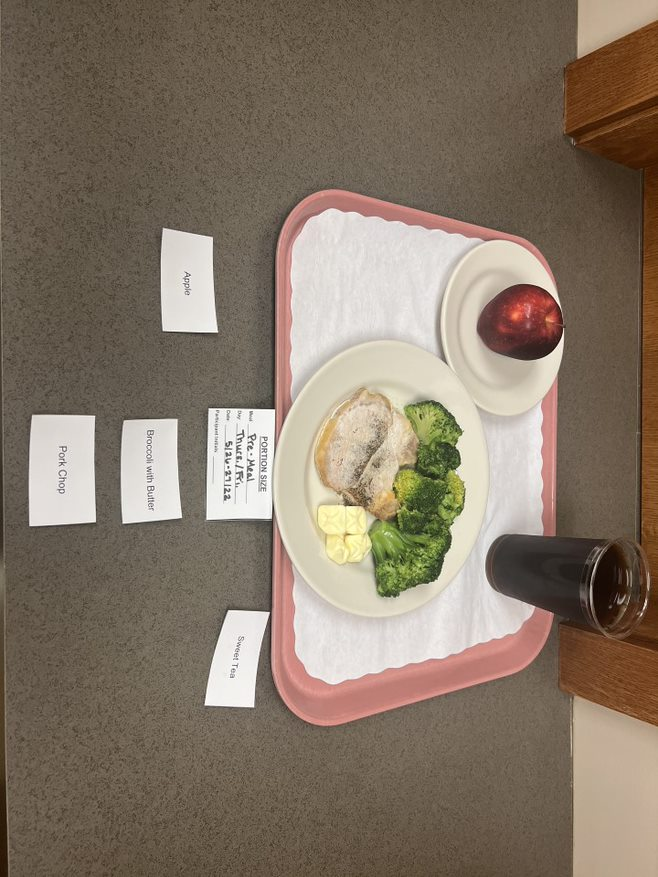


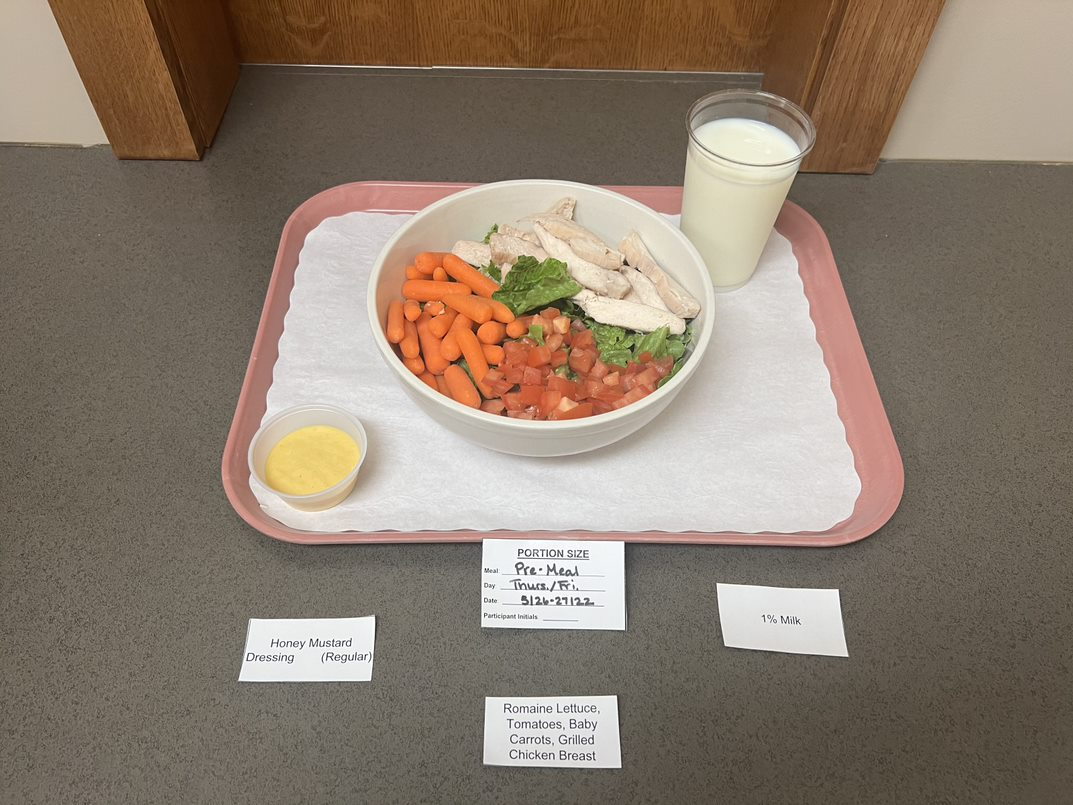


Supplementary Figure 5. Menu 5: Salad Meal (600 kcal) and Hamburger Meal (600 kcal)


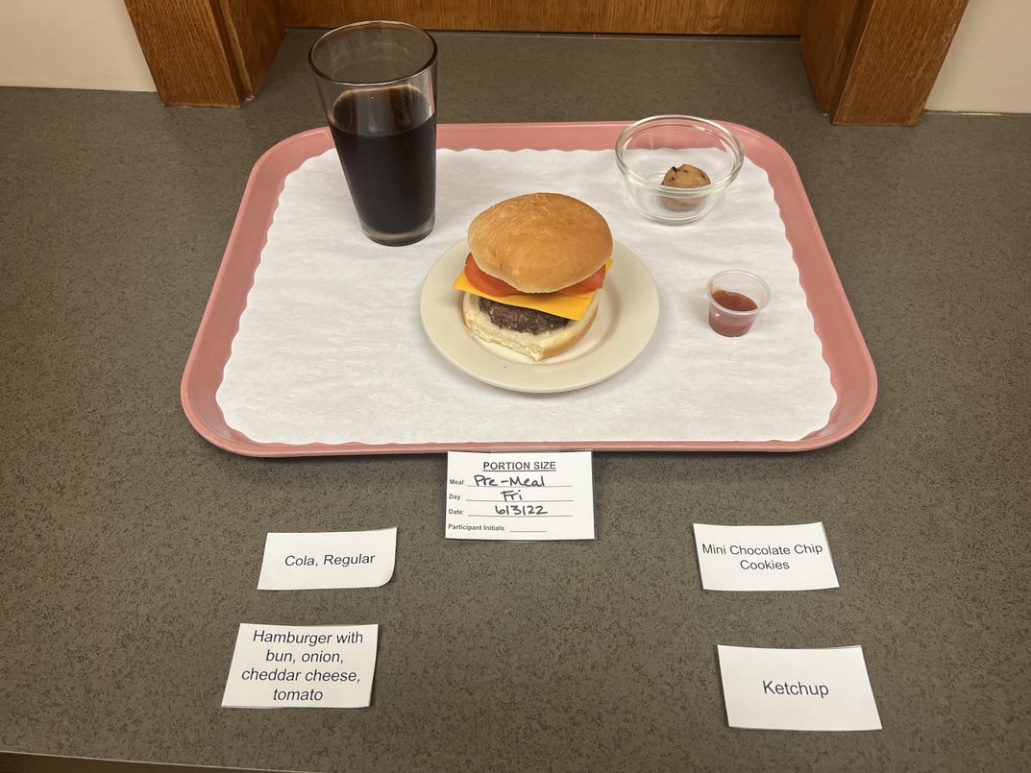


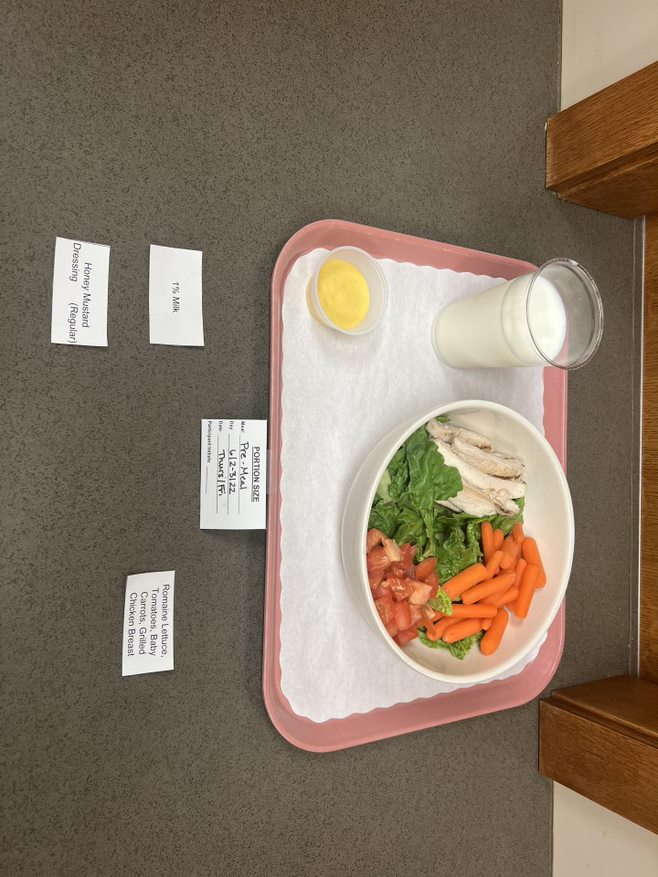


Supplementary Figure 6. General Linear Model of Automated Energy (kcal) Estimates from Openfit and Weighed Estimates.


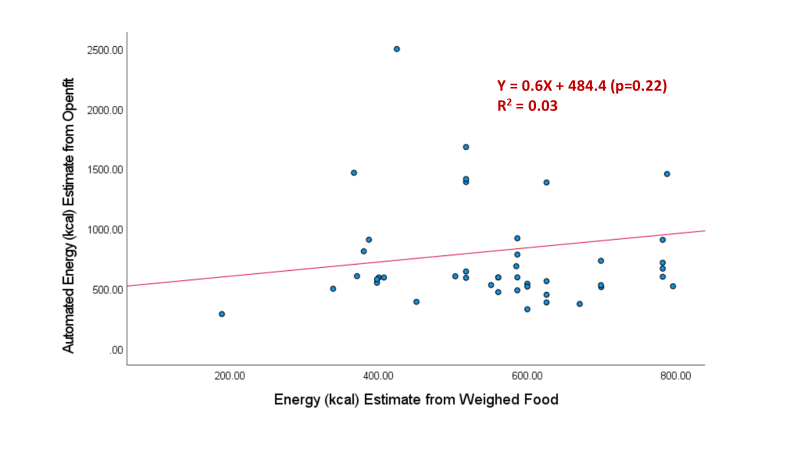


Supplementary Figure 7. General Linear Model of Semi-Automated Energy (kcal) Estimates from Openfit and Weighed Estimates.


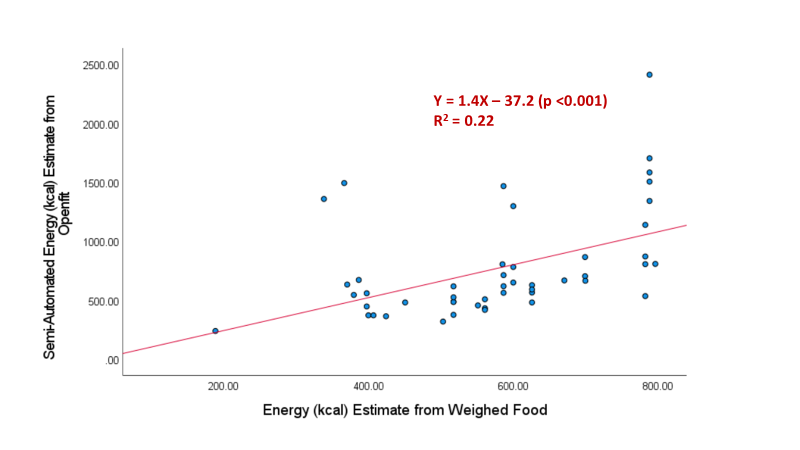


Supplementary Figure 8. General Linear Model of Automated Energy (kcal) Estimates from Openfit and Weighed Estimates, Excluding Beverages.


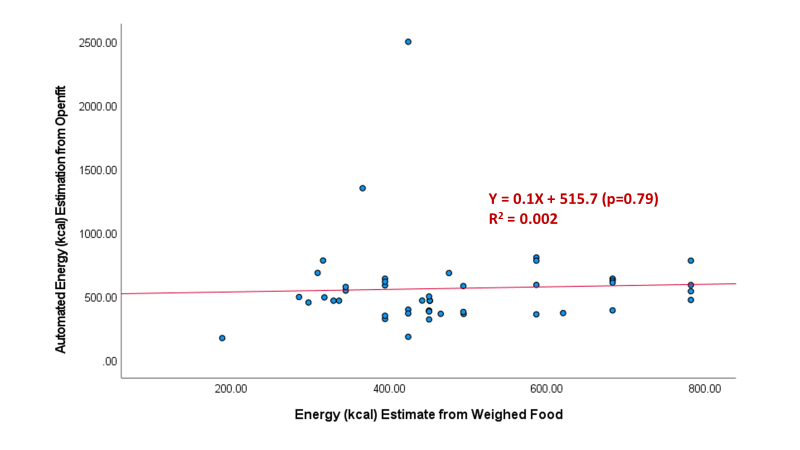


Supplementary Figure 9. General Linear Model of Automated Energy (kcal) Estimates from Openfit and Weighed Estimates, Excluding Beverages.


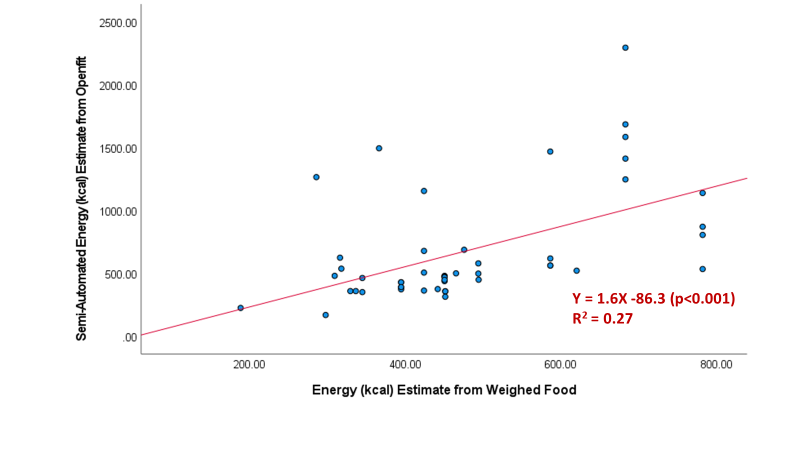

Supplement: Multimedia component1 [file mmc1.docx]
