# Supplementary material for: Validity of an Artificial Intelligence-Based Application to Identify Foods and Estimate Energy Intake Among Adults: A Pilot Study
Source: Curr Dev Nutr. 2023 Sep 29;7(11):102009. doi: 10.1016/j.cdnut.2023.102009 (PMC10656219; doi:10.1016/j.cdnut.2023.102009)

**Consort Diagram**

Title: Validity of an artificial intelligence-based app to identify foods and estimate energy intake among adults: A pilot study.

**Authors:** Chloe P. Lozano^1^, Ph.D; Emma N. Canty^1^; Sanjoy Saha^1^, Ph.D; Stephanie T. Broyles^1^, Ph.D; Robbie A. Beyl^1^, Ph.D; John W. Apolzan^1^, Ph.D; Corby K. Martin^1^, Ph.D.


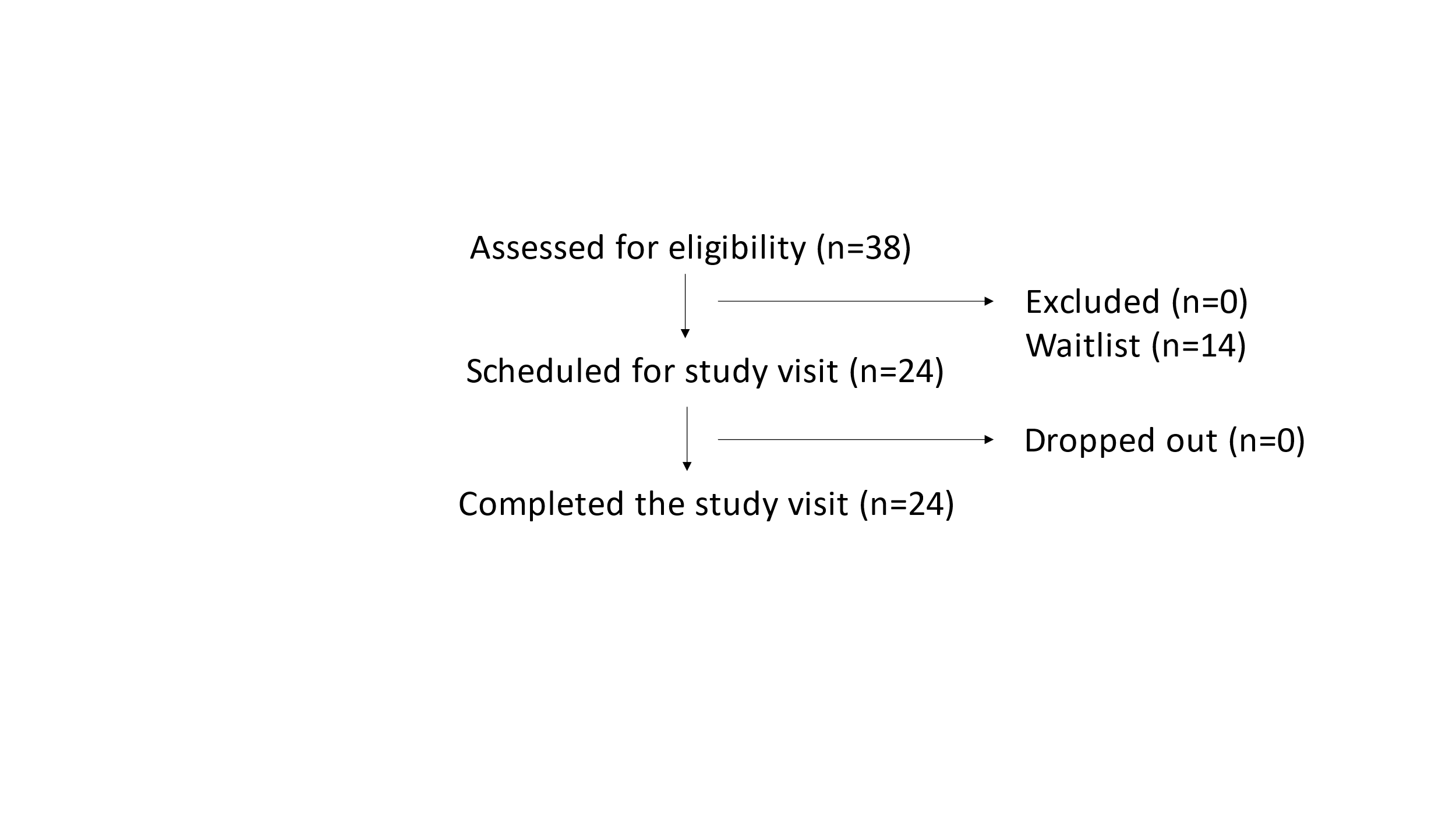

Supplement: Multimedia component2 [file mmc2.docx]
